# Non-O blood groups associated with higher risk of heart attack

**Published:** 2017

## Introduction

Having a non-O blood group is associated with a higher risk of heart attack, according to research presented recently at Heart Failure 2017 and the 4th World Congress on Acute Heart Failure.

Lead author Tessa Kole, a Master’s degree student at the University Medical Centre Groningen, the Netherlands, said: ‘It has been suggested that people with non-O blood groups (A, B, AB) are at higher risk for heart attacks and overall cardiovascular mortality, but this suggestion comes from case–control studies, which have a low level of evidence. If this was confirmed, it could have important implications for personalised medicine.’

The current study was a meta-analysis of prospective studies reporting on O and non-O blood groups, and incident cardiovascular events, including myocardial infarction (heart attack), coronary artery disease, ischaemic heart disease, heart failure, cardiovascular events and cardiovascular mortality.

The study included 1 362 569 subjects from 11 prospective cohorts, described in nine articles. There were a total of 23 154 cardiovascular events. The researchers analysed the association between blood group and all coronary events, combined cardiovascular events and fatal coronary events.

The analysis of all coronary events included 771 113 people with a non-O blood group and 519 743 people with an O blood group, of whom 11 437(1.5%) and 7 220 (1.4%) suffered a coronary event, respectively. The odds ratio (OR) for all coronary events was significantly higher in carriers of a non-O blood group, at 1.09 [95% confidence interval (CI) of 1.06–1.13].

The analysis of combined cardiovascular events included 708 276 people with a non-O blood group and 476 868 people with an O blood group, of whom 17 449 (2.5%) and 10 916 (2.3%) had an event, respectively. The OR for combined cardiovascular events was significantly higher in non-O blood group carriers, at 1.09 (95% CI 1.06–1.11).

The analysis of fatal coronary events did not show a significant difference between people with O and non-O blood groups.

'We demonstrate that having a non-O blood group is associated with a 9% increased risk of coronary events and a 9% increased risk of cardiovascular events, especially myocardial infarction’, said Ms Kole.

The mechanisms that might explain this risk are under study. The higher risk for cardiovascular events in non-O blood group carriers may be due to having greater concentrations of von Willebrand factor, a blood clotting protein which has been associated with thrombotic events. Further, non-O blood group carriers, specifically those with an A blood group, are known to have higher cholesterol. And galectin-3, which is linked to inflammation and worse outcomes in heart failure patients, is also higher in those with a non-O blood group.

Ms Kole said: ‘More research is needed to identify the cause of the apparent increased cardiovascular risk in people with a non-O blood group. Obtaining more information about risk in each non-O blood group (A, B and AB) might provide further explanations of the causes.’

She concluded: ‘In future, blood group should be considered in risk assessment for cardiovascular prevention, together with cholesterol, age, sex and systolic blood pressure. It could be that people with an A blood group should have a lower treatment threshold for dyslipidaemia or hypertension, for example. We need further studies to validate if the excess cardiovascular risk in non-O blood group carriers may be amenable to treatment.’
